# The cumulative effect of multiple high-sugar and high-fat dietary patterns on acne vulgaris in school aged children: a cross-sectional study in Shanghai

**DOI:** 10.3389/fpubh.2026.1827055

**Published:** 2026-05-08

**Authors:** Ruiqi Cai, Yan Qiang, Jinrong Lu, Mengqing Mo, Xin Ma, Fanlingzi Shen, Rui Zhang, Xiangjin Gao, Ruiping Wang

**Affiliations:** 1 Clinical Research Center, Shanghai Skin Diseases Hospital, School of Medicine, Tongji University, Shanghai, China; 2 School of Public Health, Shanghai University of Traditional Chinese Medicine, Shanghai, China; 3 Department of Traditional Chinese Medicine, Shanghai Skin Diseases Hospital, School of Medicine, Tongji University, Shanghai, China

**Keywords:** acne vulgaris, adolescent, cross-sectional study, dietary exposure index, dietary patterns

## Abstract

**Objective:**

Acne vulgaris is highly prevalent among adolescents, and diet is considered a significant modifiable associated factor. However, evidence regarding the association between popular modern beverages and adolescent acne remains limited. This study aimed to investigate the prevalence of acne and to explore the influence of the cumulative effect of multiple high-sugar and high-fat dietary patterns on acne vulgaris in school aged children in Shanghai.

**Methods:**

A cross-sectional study was conducted in 2025 in shanghai, involving 1,299 students (aged 11–16 years) among five junior high schools. Data were collected via questionnaires and onsite clinical skin examinations. A Dietary Exposure Index (DEI), ranging from 0 to 5, based on the cumulative score of five dietary items, was constructed to quantify dietary exposure to high-sugar and high-fat patterns. Statistical analyses included univariate analysis and multivariate logistic regression, and a *p-*value less than 0.05 was viewed as statistical significance.

**Results:**

The overall prevalence of acne was 64.43% (*n* = 837) in junior high school students in Shanghai, and it increased with grade level, ranging from 38.7 to 92.1% across grades. Multivariate logistic regression revealed that the intake of sugar-sweetened beverages (odds ratio [OR] = 1.56, 95% confidence interval [CI]: 1.09–2.23), milk tea (OR = 1.43, 95% CI: 1.07–1.92), and coffee (OR = 1.43, 95% CI: 1.08–1.88) was positively associated with acne vulgaris. Students with higher DEI score had a higher prevalence of acne vulgaris, and a significant dose–response relationship was observed. Multi-logistic regression indicated that students with higher DEI scores had significantly increased prevalence of acne vulgaris, the adjusted OR was 1.61 (95% CI: 0.71–3.63) for DEI = 2, 2.23 (95% CI: 1.06–4.69) for DEI = 3, 2.14 (95% CI: 1.04–4.39) for DEI = 4, and 3.12 (95% CI: 1.48–6.57) for DEI = 5, compared to the reference group (DEI = 0/1) A stronger association between higher DEI scores and acne vulgaris was also observed in younger adolescents in sub-group analysis.

**Conclusion:**

Consumption of sugar-sweetened beverages, milk tea, and coffee was associated with an increased risk of adolescent acne, and cumulative exposure to high-sugar and high-fat dietary items significantly strengthened this association. Dietary intervention strategies targeting dietary patterns in younger adolescents is important for acne prevention.

## Introduction

Acne vulgaris, a common chronic inflammatory skin disease, is characterized by lesions including comedones, papules, pustules, nodules, cysts, and potential scarring (1). It affects an estimated 9.4% of the global population, positioning it as the world’s eighth most prevalent disease. Between 1990 and 2021, the global age-standardized prevalence of acne vulgaris among population aged 10–24 year old increased from 8.56 to 9.79%. Within this spectrum, the highest prevalence is observed in adolescents aged 15–19 years, while the most marked rise occurred in those aged 10–14 years (2). As acne vulgaris is the most common disease affecting individuals aged 10–19 years (3), it represents a high-burden public health issue.

Acne vulgaris predominantly affects sebaceous-rich regions, including the face, chest, and back. The clinical presentation is primarily characterized by inflammatory papules, which can progress into nodules of varying size, often exhibiting a dusky purple to erythematous coloration. Systemic manifestations, such as fever and polyarthralgia, may occur in some cases. Inadequate management can lead to secondary pyogenic infections, and post-inflammatory scarring may persist following the resolution of active lesions (4). The significance of acne vulgaris extends beyond its prevalence, lying in its considerable impact on mental health, social functioning, economic burden and overall well-being during a crucial developmental stage. Its pathogenesis involves multiple interrelated factors, including altered follicular keratinization and differentiation, colonization by cutibacterium acnes, increased sebum production influenced by androgen activity, and immune-mediated inflammation (5).

Acne vulgaris is closely associated with dietary factors. Evidence suggests that high intake of dairy products, sugar-sweetened beverages, and foods rich in sugars and fats may elevate the risk of acne in this population (6–10). However, there remains a paucity of dedicated research examining the association between acne and increasingly popular, compositionally complex mixed beverages such as milk tea (11). Similarly, the relationship between coffee consumption and acne is not yet fully elucidated (12, 13). Therefore, in this cross-sectional study, we aimed to investigate the prevalence of acne among school-aged adolescents and to examine its associations with specific dietary factors, including milk, sugar-sweetened beverages, milk tea, coffee, and baked goods.

## Methods

### Study population

The cross-sectional study was conducted between January and May 2025. In this study, a multi-stage cluster sampling method was employed to selected school aged adolescents. Based on resource accessibility, junior high schools in seven central urban districts of Shanghai were selected as the research sites. First, three of Shanghai’s seven central urban districts were randomly selected. Subsequently, five junior high schools were randomly chosen from the three selected districts in approximating a 10% sampling rate per district (resulting in 2 schools from Jing’an, 1 from Changning, and 2 from Putuo). Finally, all students in grades 6 through 9 from the selected schools were invited to participate. Prior to questionnaire administration, the research purpose and procedures were explained to all participants. The study was approved by the Ethics Review Committee of Shanghai Skin Diseases Hospital (Approval No.2024–25).

### Sample size

In this study, the sample size was calculated using the standard formula for estimating prevalence in cross-sectional studies: *n* = [μ*_α_*^2^ × p(1-p)]/*δ*^2^. Based on a prior survey indicating an adolescent acne prevalence of approximately 42.43% in Shanghai, we set *p* = 40%, α = 0.05, and δ = 10% of p. After accounting for a design effect due to cluster sampling and an estimated 90% response rate, the minimum target sample size was determined to be 1,139 students. In this study, a total of 1,299 participants were ultimately included in the final analysis.

### Diagnosis, inclusion and exclusion criteria for acne vulgaris

In this study, the diagnosis of acne vulgaris was clinically confirmed on-site by certified dermatologists in shanghai skin disease hospital. Acne vulgaris was diagnosed based on the Chinese Guidelines for the Diagnosis and Treatment of Acne Vulgaris (14), with at least one visible lesion (comedone, papule, pustule, nodule, or cyst) confirmed by the dermatologists.

The inclusion criteria was as follows: (1) formally registered school aged adolescents in grades 6–9 in the five selected schools; (2) capable of understanding the questionnaire content; (3) provision of informed consent forms signed both by student and their guardian(s). Students with cognitive, linguistic, auditory, or visual impairments, or were absent during the survey period were excluded.

### Data collection

Data in this study was collected through a combination of a structured questionnaire and onsite clinical dermatological examination, which was administered face-to-face in these five selected schools. All qualified students anonymously completed their self-administered questionnaire under the guidance of research staff. The questionnaire covered the following areas: (1) sociodemographic features, including grade level, age, sex, ethnicity, household registration type, height, and weight; (2) self-reported clinical features of acne, encompassing disease duration, primary sites of involvement, and parental history of acne; (3) habitual intake of five dietary categories including dairy products (including type, consumption frequency, and habit duration), prepackaged sugar-sweetened beverages (carbonated drinks; consumption frequency and habit duration), milk tea (consumption frequency, preferred sweetness level, and habit duration), coffee (consumption frequency, sweetness type preference, and habit duration) and western pastries (cakes, bread, and consumption frequency).

In addition, a standardized visual skin examination was performed onsite by uniformly trained dermatologists using a standardized assessment form to evaluate and document acne status. For students clinically diagnosed with acne vulgaris, the specific types of lesions were recorded in detail, including comedones, papules, pustules, nodules, post-inflammatory erythema or hyperpigmentation, atrophic scars, and hypertrophic scars.

### Classifications, definition, index calculation

In this study, we categorized age as early adolescence (11–13 years) and mid-adolescence (14–16 years); ethnicity as Han or other ethnicity; and household registration status as local or non-local, and body mass index (BMI) as normal (<24.0 kg/m^2^), overweight (24.0–27.9 kg/m^2^), or obesity (≥28.0 kg/m^2^) according to the Chinese BMI classification criteria. Acne severity as mild (defined as the presence of comedones and/or papules only) or moderate-to-severe (defined as the presence of pustules, nodules, or cysts, with or without comedones and papules), based on the Chinese Acne Treatment Guidelines (2019 Revised Edition) and clinical practice.

The dietary assessment focused on five categories: dairy products (including milk, yogurt, and cheese), sugar-sweetened beverages (defined as prepackaged drinks with added sugar, such as carbonated beverages and fruit-flavored drinks), milk tea (commercially prepared sweetened tea-based beverages), coffee (any type of coffee preparation, irrespective of added sweeteners or dairy), and western pastries (baked goods high in refined carbohydrates and added fats, e.g., cakes and cookies). For each of the five dietary categories (dairy, sugar-sweetened beverages, milk tea, coffee, and pastries), participants were classified as exposed if they had consumed the food item within the past 2 weeks; otherwise, they were classified as unexposed. For frequency analysis, intake was further categorized into three levels: never (no consumption within 2 weeks), sometimes (irregular consumption, i.e., consumed but not daily), and daily (consumed at least once per day).

To assess cumulative dietary exposure, a Dietary Exposure Index (DEI) was constructed in this study. The DEI ranged from 0 to 5, with one point assigned for each of the five dietary categories where the participant was classified as exposed (exposed = 1, unexposed = 0). In this study, duo to the unexposed group (DEI = 0) included only 4 participants, which was too small for stable statistical analysis, so it was merged with the low-exposure group (DEI = 1, *n* = 35) to form the reference group (DEI = 1, *n* = 39, 3.1%) in the subsequent logistic regression analysis.

### Statistical analysis

In this study, statistical analyses were performed using SAS 9.4. Quantitative data are presented as mean and standard deviation (SD) or median (interquartile range, IQR), and group comparisons were conducted using the independent-samples *t*-test or the Mann–Whitney *U* test, as appropriate. Qualitative data are expressed as frequency (percentage), with comparisons made by the chi-square test. To explore the associations between dietary factors (DEI) and the prevalence of acne vulgaris in school aged adolescents, the logistic regression (LR) was applied to calculate the odds ratio (OR) and 95% confidence interval (CI), with the adjustment of age, gender, ethinic, hukou status, family history and BMI. In the Model B of logistic regression, all available covariates (age, BMI, gender, ethnicity, household registration, family history) were incorporated for adjustment. In the Model C of logistic regression, we only retained variables with *p* < 0.05 from Model B (age, BMI categories) for adjustment. In this study, a two-sided *p-*value < 0.05 was considered as statistically significant.

## Results

In this study, 1,299 students aged 11–16 years were recruited, with a median age of 13 years (interquartile range: 13–14 years). The mean value and corresponding SD for height, body weight, and BMI were 164.0 (8.9) cm, 53.9 (13.1) kg, and 19.9 (4.0) kg/m^2^, respectively. Moreover, the number of students in grade 6, 7, 8 and 9 was 364 (28.0%), 328 (25.3%), 404 (31.1%), 203 (15.6%), respectively. The majority were of male (*n* = 688, 53.0%), Han ethnicity (*n* = 1,272, 97.9%), and 830 (63.9%) possessed local household registration (Hu Kou status). In this study, 837 (64.4%) students were diagnosed with acne vulgaris. Students in the acne group (median age 14 years, *n* = 837) were slightly older than those in the healthy group (median age 13 years, *n* = 462) (*p* < 0.001). Students in the acne group had significantly greater height, weight, and BMI than those in healthy group (Table 1).

**Table 1:** Baseline characteristics of school-aged adolescents in Shanghai by acne status.

Characteristics	Total (*n* = 1,299)	Acne (*n* = 837)	Healthy (*n* = 462)	*P*-value
Age(years), median (P25, P75)	13 (13,14)	14 (13,14)	13 (12,13)	0.000
Age group, *n* (%)				0.000
≤13 years	715 (55.0)	343 (48.0)	372 (52.0)	
>13 years	584 (45.0)	494 (84.6)	90 (15.4)	
Height (cm), mean (SD)	164.0 (8.9)	165.8 (8.4)	160.6 (8.7)	0.000
Weight (kg), mean (SD)	53.9 (13.1)	56.0 (13.1)	50.1 (12.2)	0.000
BMI (kg/m^2^), mean (SD)^a^	19.9(4.0)	20.2(3.9)	19.3(4.0)	0.000
BMI group, *n* (%)				0.004
N**ormal (<24.0)**	1,179 (90.8)	743 (63.0)	436 (37.0)	
Overweight (24.0–27.9)	77 (5.9)	60 (77.9)	17 (22.1)	
Obesity (>28.0)	43 (3.3)	34 (79.1)	9 (20.9)	
Grade, *n* (%)				0.000
Six	364 (28.0)	141 (38.7)	223 (61.3)	
Seven	328 (25.3)	174 (53.0)	154 (47.0)	
Eight	404 (31.1)	335 (82.9)	69 (17.1)	
Nine	203 (15.6)	187 (92.1)	16 (7.9)	
Gender, *n* (%)				0.396
Male	688 (53.0)	436 (63.4)	252 (36.6)	
Female	611(47.0)	401 (65.6)	210 (34.4)	
Ethnicity, *n* (%)^b^				0.515
Han	1,272 (97.9)	818 (64.3)	454 (35.7)	
Other Ethnicity	27 (2.1)	19 (70.4)	8 (29.6)	
Hu Kou status, *n* (%)^c^				0.000
Local	830 (63.9)	563 (67.8)	267 (32.2)	
Non-local	469 (36.1)	274 (58.4)	195 (41.6)	
Family history, *n* (%)				0.266
Yes	339 (26.1)	210 (61.9)	129 (38.1)	
No	960 (73.9)	627 (65.3)	333 (34.7)	

SD, standard deviation; BMI, body mass index. ^a^ BMI was calculated as weight in kilograms divided by height in meters squared. ^b^ Ethnicity was classified according to the standards of the National Bureau of Statistics of China. The Han ethnicity (the majority ethnic group in China) served as the reference group. “Other Ethnicity” includes 55 officially recognized ethnic minority groups (e.g., Zhuang, Hui, Manchu, Uygur, Miao, etc.). ^c^ Hu Kou status: Refers to China’s household registration system. In this study, “Local” indicates possession of a Shanghai household registration; “Non-local” indicates registration in another city or region within China.

### Association between five dietary items exposure and adolescent acne vulgaris

In this study, students with acne vulgaris had higher proportion of sugar sweetened beverage (88.6% vs. 84.0%), milk tea (79.4% vs. 74.0%) and coffee (36.6% vs. 24.9%) than those without acne, the differences were statistically significant (*p* < 0.05). Moreover, a significant dose–response relationship was observed for the DEI and acne prevalence (Cochran-Armitage trend test, *Z* = 3.348, *p* < 0.05), demonstrating an elevated acne prevalence with higher DEI value (Table 2).

**Table 2:** Dietary exposures of school-aged adolescents in shanghai by acne status.

**Dietary exposure**	**Total (*n* = 1,299)**	**Acne (*n* = 837)**	**Healthy (*n* = 462)**	***P-*value**
Dairy products, *n* (%)				0.647
Yes	1,209 (93.1)	777 (92.8)	432 (93.5)	
No	90 (6.9)	60 (7.2)	30 (6.5)	
SSB, *n* (%)				0.017
Yes	1,130 (87.0)	742 (88.6)	388 (84.0)	
No	169 (13.0)	95 (11.4)	74 (16.0)	
Milk tea, *n* (%)				0.033
Yes	1,005 (77.4)	663 (79.2)	342 (74.0)	
No	294 (22.6)	174 (20.8)	120 (26.0)	
Coffee, *n* (%)				0.000
Yes	432 (32.4)	306 (36.6)	115 (24.9)	
No	878 (67.6)	531 (63.4)	347 (75.1)	
Bakery products, *n* (%)				0.995
Yes	1,133 (87.2)	730 (87.2)	403 (87.2)	
No	166 (12.8)	107 (12.8)	59 (12.8)	
DEI, *n* (%)^a^				0.000 ^b^
1	39 (3.0)	19 (48.7)	20 (51.3)	
2	103 (7.9)	59 (57.3)	44 (42.7)	
3	291 (22.4)	190 (65.3)	101 (34.7)	
4	546 (42.0)	332 (60.8)	214 (39.2)	
5	320 (24.6)	237 (74.1)	83 (25.9)	

SSB, Sugar-Sweetened Beverages. ^a^ DEI (Dietary Exposure Index): Cumulative score (0–5) for five items: dairy products, sugar-sweetened beverages, milk tea, coffee, and bakery products (1 point per item). DEI = 0 (*n* = 4) was merged into the DEI = 1 group to form the low-exposure reference. ^b^ The association between Dietary Exposure Index and acne status was assessed using the Pearson chi-square test (*χ*^2^ = 22.70, *P* < 0.001). A significant dose–response trend was further evaluated using the Cochran–Armitage trend test (*Z* = 3.348, two-sided *p* < 0.001).

As shown in Table 3, the intake of sugar-sweetened beverages (SSB) (OR = 1.49, 95% CI: 1.07–2.07), milk tea (OR = 1.34, 95% CI: 1.02–1.75), and coffee (OR = 1.74, 95% CI: 1.34–2.24) were significantly associated with increased risk of acne (Model A). In Model C (with the adjusted for age, BMI and family history of acne), the significant associations persisted for SSB (OR = 1.56, 95% CI: 1.09–2.23), milk tea (OR = 1.43, 95% CI: 1.07–1.92), and coffee (OR = 1.43, 95% CI: 1.08–1.88).

**Table 3:** Association between acne and dietary exposure by each type of food in school aged children.

Dietary exposure type	n (%)	Model A		Model B		Model C	
		OR (95% CI)	P	OR (95% CI)	P	OR (95% CI)	P
Milk							
Unexposed	90 (6.9)	Ref		Ref		Ref	
Exposed	1,209 (93.1)	0.90 (0.57–1.42)	0.647	0.99 (0.60–1.63)	0.967	0.99 (0.60–1.62)	0.959
Milk intake frequency							
Never	90 (6.9)	Ref		Ref		Ref	
Sometimes	615 (47.4)	0.94 (0.59–1.51)	0.808	1.01 (0.60–1.69)	0.979	1.01 (0.60–1.68)	0.975
Daily	594 (45.7)	0.86 (0.54–1.37)	0.516	0.97 (0.58–1.63)	0.916	0.97 (0.58–1.61)	0.895
SSB							
Unexposed	169 (13.0)	Ref		Ref		Ref	
Exposed	1,130 (87.0)	**1.49 (1.07–2.07)**	**0.017**	**1.55 (1.08–2.23)**	**0.018**	**1.56 (1.09–2.23)**	**0.016**
SSB intake frequency							
Never	169 (13.0)	Ref		Ref		Ref	
Sometimes	987 (76.0)	**1.48 (1.06–2.06)**	**0.021**	**1.54 (1.07–2.22)**	**0.021**	**1.55 (1.08–2.23)**	**0.019**
Daily	143 (11.0)	1.59 (1.00–2.53)	0.049	1.63 (0.99–2.70)	0.057	1.64 (0.99–2.71)	0.054
Milk tea							
Unexposed	294 (22.6)	Ref		Ref		Ref	
Exposed	1,005 (77.4)	**1.34 (1.02–1.75)**	**0.033**	**1.41 (1.05–1.89)**	**0.024**	**1.43 (1.07–1.92)**	**0.016**
Milk tea intake frequency							
Never	294 (22.6)	Ref		Ref		Ref	
Sometimes	966 (74.4)	**1.35 (1.03–1.76)**	**0.029**	**1.42 (1.05–1.91)**	**0.021**	**1.44 (1.08–1.93)**	**0.015**
Daily	39 (3.0)	1.10 (0.56–2.19)	0.778	1.14 (0.53–2.45)	0.740	1.21 (0.57–2.56)	0.619
Coffee							
Unexposed	878 (67.6)	Ref		Ref		Ref	
Exposed	432 (32.4)	**1.74 (1.34–2.24)**	**0.000**	**1.43 (1.09–1.89)**	**0.011**	**1.43 (1.08–1.88)**	**0.011**
Coffee intake frequency							
Never	878 (67.6)	Ref		Ref		Ref	
Sometimes	377 (29.0)	**1.74 (1.34–2.26)**	**0.000**	**1.50 (1.12–1.99)**	**0.006**	**1.48 (1.12–1.97)**	**0.007**
Daily	44 (3.4)	1.74 (0.89–3.43)	0.108	0.93 (0.43–1,98)	0.841	0.97 (0.46–2.04)	0.929
Bakery							
Unexposed	166 (12.8)	Ref		Ref		Ref	
Exposed	1,133 (87.2)	1.00 (0.71–1.40)	0.995	1.19 (0.82–1.73)	0.357	1.19 (0.82–1.73)	0.364
Bakery intake frequency							
Never	166 (12.8)	Ref		Ref		Ref	
Sometimes	989 (76.1)	1.06 (0.75–1.49)	0.751	1.26 (0.86–1.84)	0.234	1.26 (0.86–1.84)	0.233
Daily	144 (11.1)	0.69 (0.44–1.09)	0.111	0.84 (0.51–1.38)	0.486	0.82 (0.50–1.35)	0.433

OR, odds ratio; CI, confidence interval; SSB, Sugar-sweetened beverages. Model A: Univariate logistic regression assessing the unadjusted association between each food and acne. Model B: Multivariable logistic regression with all variables incorporated into the model (age, BMI, gender, ethnicity, household registration status, acne history). Model C: Multivariable logistic regression with the adjustment of age, BMI and family history. Bold values indicate odds ratios and p-values that are statistically significant (uncorrected p < 0.05 for comparisons that do not require correction; Bonferroni-corrected *p* < 0.025 for multiple comparisons).

**Figure 1:**
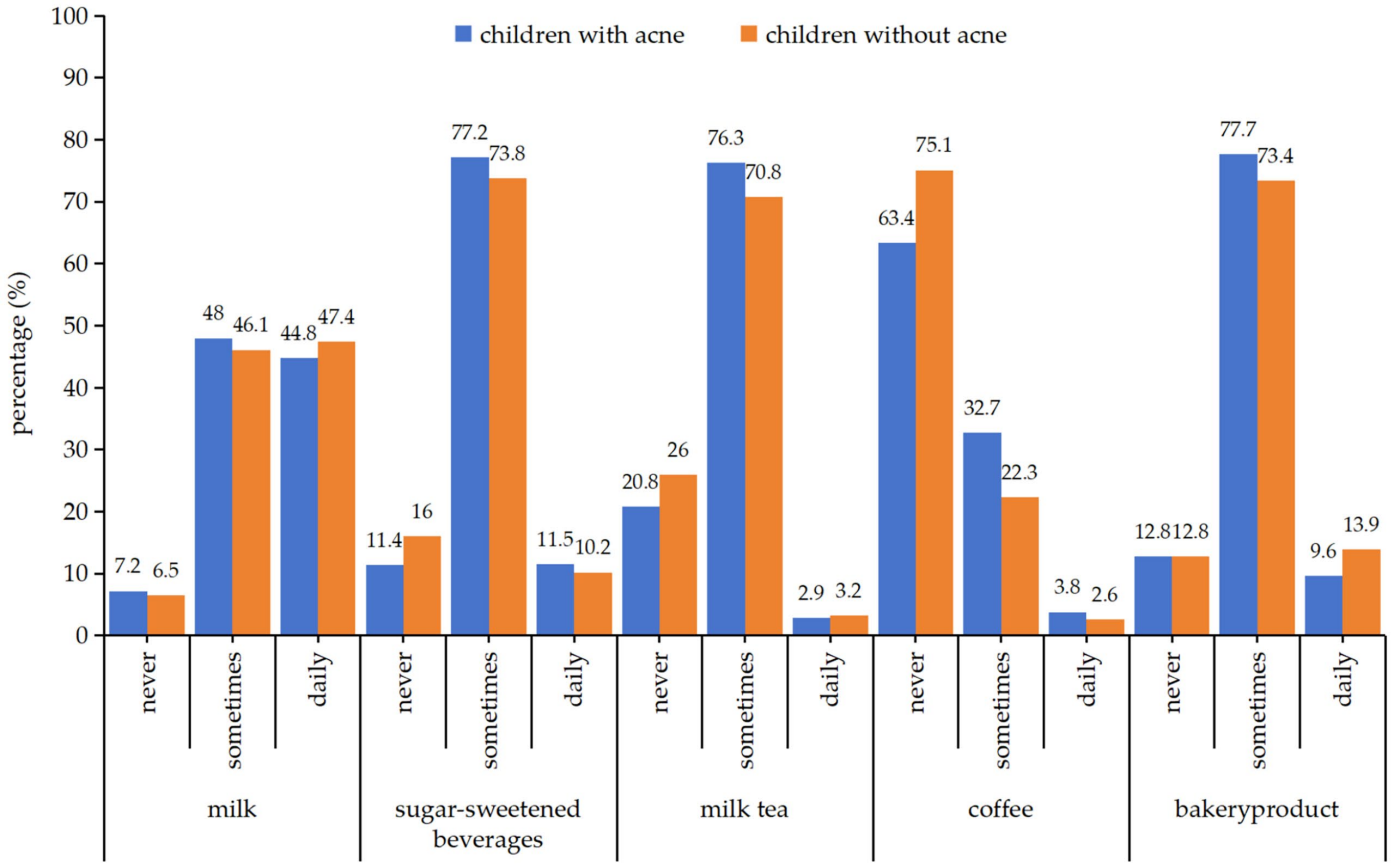
Frequency of dietary exposures among school-aged adolescents in Shanghai with and without acne.

In this study, we also explored the association between intake frequency of the five dietary items and the prevalence of acne vulgaris. The intake frequency patterns (never, sometimes and daily) for milk, sugar-sweetened beverages (SSB), milk tea, and bakery products were comparable between students with and without acne vulgaris, with “sometimes” pattern being the predominant frequency reported. However, students with acne were more likely to drink coffee, with a higher proportion of both “sometimes” (32.7% vs. 22.3%) and “daily” (3.8% vs. 2.6%) coffee consumption compared to those without acne (Figure 1).

In Table 3, multivariable logistic regression analysis adjusting for age, BMI, and family history showed that, compared with never intake, students with ‘sometimes’ and ‘daily’ intake of sugar-sweetened beverages (SSB), milk tea, and coffee had higher odds of acne vulgaris. Specifically, for SSB, the odds ratios (ORs) were 1.55 (95% CI: 1.08–2.23) for ‘sometimes’ and 1.64 (95% CI: 0.99–2.71) for ‘daily’; for milk tea, the ORs were 1.44 (95% CI: 1.08–1.93) for ‘sometimes’ and 1.21 (95% CI: 0.57–2.56) for ‘daily’; for coffee, the OR for ‘sometimes’ intake was 1.48 (95% CI: 1.12–1.97) (Table 3).

### Association between dietary exposure index and adolescent acne vulgaris

In this study, students over 13 years were more likely to have acne vulgaris than those aged 13 years or younger, the OR was 5.95, (95%CI:4.55–7.78). Compared with normal BMI (<24.0), overweight (24.0–27.9) and obesity (≥28.0) were both associated with increased odds of acne (OR = 2.07, 95% CI: 1.19–3.60 and OR = 2.22, 95% CI: 1.05–4.67, respectively). Students with non-local residency status had a significantly lower odds of acne compared to those with local residency status (OR = 0.67, 95%CI:0.53–0.84). For the Dietary Exposure Index (DEI), students with higher DEI score had higher prevalence of acne vulgaris, and taking the low-exposure group (DEI = 1) as the reference, significantly higher odds of acne were observed at specific exposure levels: for DEI 3 (OR = 1.98, 95% CI:1.01–3.88) and for DEI 5 (OR = 3.01, 95% CI: 1.53–5.91) (Table 4).

**Table 4:** Multivariable logistic regression analysis of the association between dietary exposure and acne.

Variable	Model A		Model B		Model C	
	OR (95%CI)	*P*	OR (95%CI)	*P*	OR (95%CI)	*P*
Age						
≤13 years	Ref ^a^		Ref		ref	
>13 years	**5.95 (4.55–7.78)**	**0.000**	**5.78 (4.39–7.62)**	**0.000**	**5.75 (4.37–7.58)**	**0.000**
BMI group						
Normal (<24.0)	Ref		Ref		Ref	
Overweight (24.0–27.9)	**2.07 (1.19–3.60)**	**0.001**	**2.13 (1.17–3.85)**	**0.013**	**2.08 (1.15–3.74)**	**0.015**
Obesity (>28.0)	2.22 (1.05–4.67)	0.036	1.76 (0.79–3.94)	0.167	1.70 (0.77–3.79)	0.192
Gender						
Male	Ref		Ref			
Female	1.10 (0.88–1.39)	0.396	1.11 (0.86–1.43)	0.434		
Ethnicity						
Han	Ref		Ref			
Other Ethnicity	1.32 (0.57–3.04)	0.516	1.08 (0.43–2.71)	0.870		
Hu Kou status						
Local	Ref		Ref			
Non-local	**0.67 (0.53–0.84)**	**0.001**	0.89 (0.68–1.14)	0.322		
Family history						
No	Ref		Ref			
Yes	1.16 (0.90–1.50)	0.266	0.76 (0.57–1.02)	0.064		
DEI ^c^						
1	**Ref**		**Ref**		**Ref**	
2	1.41 (0.67–2.96)	0.361	1.71 (0.75–3.87)	0.201	1.61 (0.71–3.63)	0.256
3	1.98 (1.01–3.88)	0.047	2.35 (1.12–4.95)	0.025	2.23 (1.06–4.69)	0.035
4	1.63 (0.85–3.13)	0.140	2.20 (1.07–4.53)	0.032	2.14 (1.04–4.39)	0.039
5	**3.01 (1.53–5.91)**	**0.001**	**3.22 (1.53–6.80)**	**0.002**	**3.12 (1.48–6.57)**	**0.003**

OR, odds ratio; CI, confidence interval; DEI, dietary exposure index. ^a^ Denotes the reference group, for which the odds ratio (OR) is defined as 1. ^b^ BMI was calculated as weight in kilograms divided by height in meters squared. ^c^ DEI (Dietary Exposure Index): Cumulative score (0–5) for five items: dairy products, sugar-sweetened beverages, milk tea, coffee, and bakery products (1 point per item). DEI = 0 (*n* = 4) was merged into the DEI = 1 group to form the low-exposure reference. Model A: Univariate logistic regression assessing the unadjusted association between each variable and acne. Model B: Multivariable logistic regression with all variables incorporated into the model (age, BMI, gender, ethnicity, Hukou status, Family history and DEI). Model C: Multivariable logistic regression with the adjustment of age and BMI. Bold values indicate odds ratios and *p*-values that are statistically significant after Bonferroni correction for multiple comparisons.

In Model B, which included all available covariates (age, BMI, gender, ethnicity, household registration, family history) for adjustment, findings indicated that students with DEI value of 3, 4, and 5 had substantially elevated odds at these higher exposure levels. The OR was 1.71 (95% CI:0.75–3.87) for DEI 2, 2.35 (95% CI:1.12–4.95) for DEI 3, 2.20 (95% CI:1.07–4.53) for DEI 4, an 3.22 (95% CI:1.53–6.80) for DEI 5. Further, Model C retained only variables with *p* < 0.05 from Model B (age, BMI categories) for adjustment, findings also revealed higher odds of acne prevalence for DEI 2 (OR = 1.61,95% CI:0.71–3.63), DEI 3 (OR = 2.23, 95% CI:1.06–4.69), DEI 4 (OR = 2.14, 95% CI:1.04–4.39), and DEI 5 (OR = 3.12, 95% CI:1.48–6.57) compared to the low-exposure group (DEI 1) (Table 4).

### DEI and acne vulgaris association among students in different age group

In this study, subgroup analysis of the association between DEI and acne vulgaris indicated significant dose–response relationship among students aged <13 years, with higher DEI value associated with increased odds of acne vulgaris. The OR was 2.84 (95% CI:0.83–9.70) for DEI 2, 3.12 (95% CI:0.98–9.88) for DEI 3, 3.22 (95% CI:1.05–9.92) for DEI 4, and 5.69 (95% CI:1.79–18.05) for DEI 5, respectively. In contrast, in the age group of 14–16 years, none of the DEI categories (2–5) showed a statistically significant association with acne compared with the reference group (all *p* > 0.05) (Table 5).

**Table 5:** The association between dietary exposure and acne by different age group.

Group	11–13 years		14–16 years	
	OR (95%CI)	*P-*value	OR (95%CI)	*P-*value
BMI group				
Normal (<24.0)	Ref		Ref	
Overweight (24.0–27.9)	**2.18 (1.11–4.30)**	**0.024**	3.50 (0.77–16.00)	0.106
Obesity (≥28.0)	1.68 (0.62–4.56)	0.308	2.25 (0.50–10.14)	0.290
Gender				
Male	Ref		Ref	
Female	1.32 (0.97–1.80)	0.076	0.81 (0.50–1.31)	0.388
Ethnicity				
Han	Ref		Ref	
Other Ethnicity	3.51 (0.91–13.57)	0.069	**0.29 (0.09–0.92)**	**0.037**
Hu Kou status				
Local	Ref		Ref	
Non-local	1.18 (0.87–1.62)	0.285	**0.40 (0.25–0.64)**	**0.000**
Family history				
No	Ref		Ref	
Yes	0.65 (0.45–0.93)	0.019	1.16 (0.67–1.99)	0.595
DEI				
1	Ref		Ref	
2	2.84 (0.83–9.70)	0.096	0.79 (0.21–3.01)	0.732
3	3.12 (0.98–9.88)	0.054	1.59(0.46–5.49)	0.465
4	3.22 (1.05–9.92)	0.042	1.44 (0.44–4.76)	0.550
5	**5.69 (1.79–18.05)**	**0.003**	1.68 (0.50–5.63)	0.404

OR, odds ratio; CI, confidence interval; DEI, dietary exposure index; BMI, body mass index. Multivariable logistic regression models were constructed, respectively, for adolescents aged 11–13 years and 14–16 years. All models were adjusted for body mass index (BMI), gender, ethnicity, household registration (Hu Kou) status, and family history. Bold values indicate odds ratios and *p*-values that are statistically significant after Bonferroni correction for multiple comparisons.

**Figure 2:**
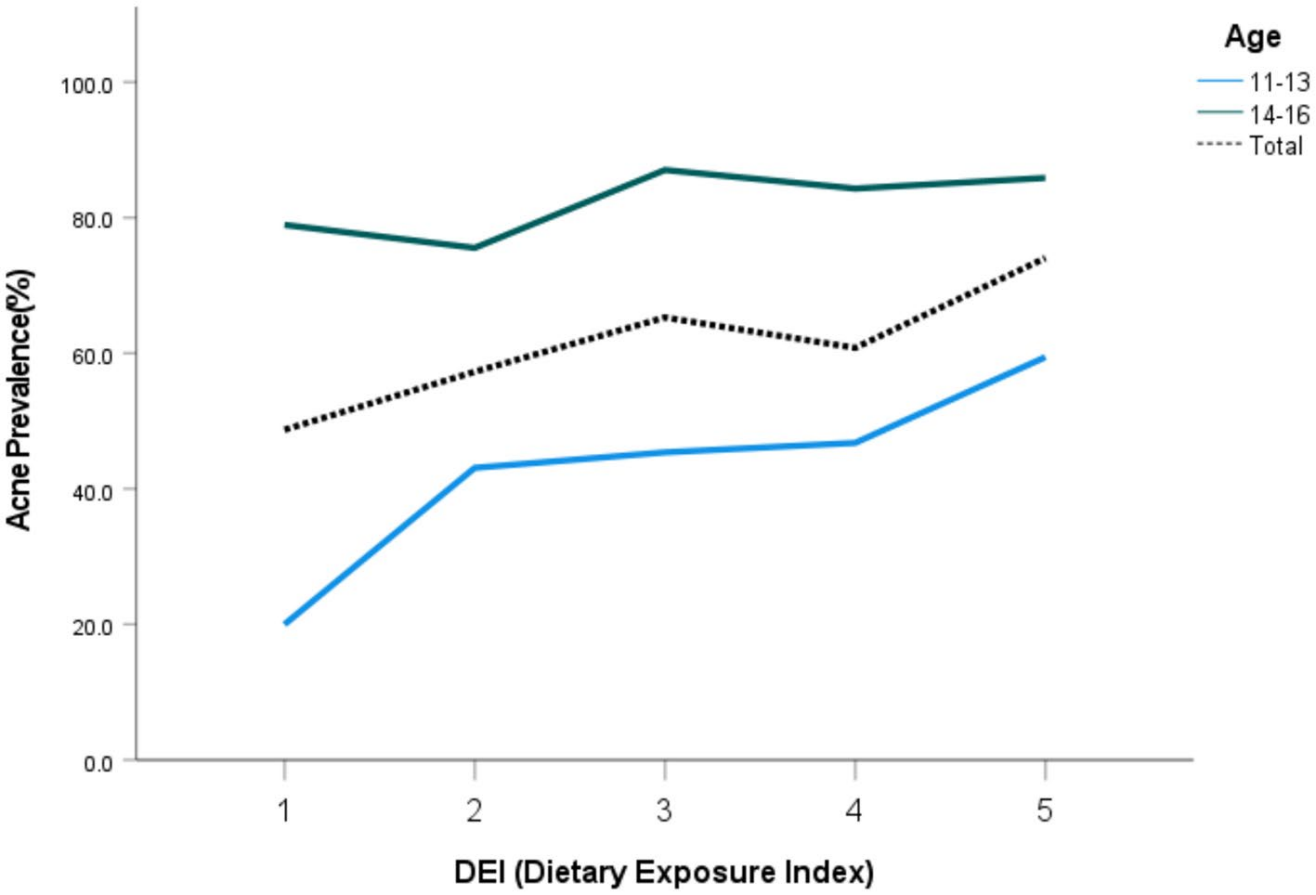
Prevalence of acne by DEI score and age group among school-aged adolescents in Shanghai.

Furthermore, at all DEI levels, acne prevalence was consistently higher in the 14–16 years group than in the 11–13 years group. Notably, within each age stratum and total school-aged adolescents, the prevalence exhibited an increasing trend with higher Dietary Exposure Index (DEI) levels (Figure 2).

### The association between DEI and severity of acne vulgaris among students

In this study, among the 837 students with acne vulgaris, 624 (74.6%) had mild acne, and 213 (25.4%) had moderate-to-severe (MS) acne, the prevalence rate was 48.0% for mild acne, and 16.4% for MS acne. Students with MS acne were older, had higher BMI value and had a higher proportion of acne family history (Supplementary Table S1). Students who consumed sugar-sweetened beverage had higher proportion of mild acne than those who did not (49.0% vs. 40.2%). Conversely, students who consumed milk tea (17.8% vs. 11.6%) or coffee (17.2% vs. 12.1%) had a higher proportion of MS acne. Regarding the DEI, students with a DEI of 2–5 had a markedly higher proportion of MS acne than students with a DEI of 1 (Supplementary Table S2).

Ordered multivariable logistic regression analysis of acne vulgaris severity (none, mild, moderate-to-severe), adjusting for age, BMI, gender, ethnicity, and family history of acne, showed that a higher DEI value was associated with higher odds of acne vulgaris prevalence. Specifically, compared with a DEI of 0 or 1 (reference), the odds ratios were 1.97 for DEI = 2, 2.60 for DEI = 3 or 4, and 3.25 for DEI = 5 (Supplementary Figure S1).

## Discussion

This study examined the relationship between modern beverage consumption, cumulative dietary patterns, and adolescent acne. The results indicated that elevated BMI, older age, and the intake of sugar-sweetened beverages, milk tea, and coffee were all associated with an increased odds of acne in school-aged adolescents, although the effect sizes of the individual dietary exposures were modest. Further analysis indicated a positive correlation between the number of DEI and acne vulgaris prevalence, which was particularly pronounced in the younger cohort (11–13 years).

In this study, the mean BMI value was significantly higher in the acne group compared to those without acne, indicating that the elevated BMI is associated with an increased odds of adolescent acne. This finding is consistent with prior research, including a cross-sectional study in Nigerian secondary schools which reported that adolescents with higher BMI were more likely to have facial acne vulgaris, although acne severity itself was not correlated with BMI (15). This study also aligns with a survey from Sudan, which similarly reported that acne prevalence correlated positively with both age and higher BMI in adolescent (16). However, conclusions across different studies remain inconsistent. A large-scale cohort study based on conscription data demonstrated an inverse relationship between severe obesity and acne after multivariate adjustment (males: aOR = 0.53, 95% CI:0.42–0.64; females: aOR = 0.50, 95% CI:0.37–0.62) (17). These discrepancies may be attributed to ethnic differences and the military setting of their study. Currently, there is no definitive consensus on the association between BMI and acne, and the precise underlying biological mechanisms have not been fully elucidated. Existing hypotheses propose that obesity is linked to acne via adipose tissue: increased adiposity may promote elevated androgen synthesis, which in turn stimulates sebum overproduction, a key factor in acne pathogenesis (18). This finding implies that the association between BMI and acne may be mediated via hormonal and metabolic pathways. Further investigations are thus warranted to unravel the precise underlying mechanisms and clarify the heterogeneous patterns observed across distinct populations.

Consistent with findings of preceding studies, the present investigation also demonstrated an association between sugar-sweetened beverage consumption and the occurrence of acne among adolescents (9, 10). A plausible underlying mechanism is that diets high in sugar and fat may activate the insulin/insulin-like growth factor-1 (IGF-1) signaling pathway, thereby enhancing sebum synthesis and exacerbating cutaneous inflammatory responses (8). Although the observed association between dairy intake and acne did not attain statistical significance in this study, which stands in contrast to the evidence reported in a meta analysis by Christian R. Juhl et al. (7). One potential rationale for this observation lies in the very high consumption prevalence of milk (93.1%) among students in Shanghai; this limited variability in exposure status undermined the statistical power required for effective differentiation between milk consumer and non-consumer groups, ultimately hindering the identification of a statistically independent association within this study sample. The association between milk tea consumption and acne identified in this study represents a suggestive finding. Milk tea has a complex composition, characterized by high levels of sugar and fat, and potentially trans-fatty acids (11, 19). Its association may stem from a synergistic effect of these multiple components. Regarding coffee, the findings of this study suggest an association with an increased odds of acne. This may be explained by the common practice of adding sugar to coffee, which could potentially offset the anti-inflammatory effects of caffeine (12, 13). Given that milk tea and coffee are often consumed with added sugar, their observed association with acne might be confounded by sugar intake. Future studies should aim to disentangle the effects of the beverage base from added sweeteners. Further more, the occasional intake of sugar-sweetened beverages (SSB), milk tea, and coffee was consistently associated with higher odds of acne, while daily intake of these items did not reach statistical significance in the fully adjusted models. The lack of significance for daily consumption is likely due to the small sample sizes of the daily intake subgroups (SSB, *n* = 143; milk tea, *n* = 39; coffee, *n* = 44), which might limited the statistical power to detect modest effects.

In this study, analysis using the DEI suggested a positive dose–response relationship, with higher DEI value generally associated with increased acne risk. These findings indicate that acne development might be more likely driven by the combined effect of multiple dietary factors, rather than any single component, with various high-sugar and high-fat foods potentially acting through common biological mechanisms. The core mechanism likely involves synergistic activation of insulin/IGF-1 signaling axis by these dietary components, which promotes sebum hyper-secretion and follicular hyper-keratinization, ultimately leads to acne development (20). Future research should validate these associations by expanding the sample size and balancing the distribution across exposure groups.

In the study, pubertal stages were operationally defined based on age, where the early pubertal stage was designated as 11–13 years and the mid-pubertal stage as 14–16 years. This grouping was anchored in a core endocrine hallmark of puberty, thus highlighting the physiological distinction between preandrogen peak phase (11–13 years) and androgen peak phase (14–16 years) (21). In this study, subgroup analysis by age revealed a distinct pattern. A higher DEI value was positively associated with the odds of acne among younger students (11–13 years). This association was markedly attenuated in older adolescents (14–16 years), a group that notably exhibited a higher prevalence of acne. This pattern may be linked to endocrine changes during adolescence. Hormonal profiles, including serum insulin-like growth factor-1 (IGF-1), exhibit a peak concentration in the mid-pubertal stage (22). This powerful endogenous driver may thus become the key contributor to acne pathogenesis, thereby reducing the relative influence of exogenous factors like diet. The present finding aligns well with previously documented epidemiological trends, which demonstrate that the incidence of acne exhibits the fastest growth rate between the ages of 10 and 14 (2). Collectively, the results of this study indicate that delivering dietary interventions tailored specifically to younger adolescents in the early pubertal stage could carry considerable significance for the prevention of acne.

### Limitations

This study has several limitations. First, as the sample was drawn from junior high school students in Shanghai, the generalizability of the findings may be limited, particularly to those in other regions or age groups. Second, as a cross-sectional survey in which dietary information was collected through face-to-face interviews, recall bias may exist and the design itself does not allow for causal conclusions. Third, this study was unable to assess the specific intake amounts or sugar content of individual dietary items. Additionally, potential lifestyle confounders such as physical exercise, sleep duration, skincare behavior, overall dietary patterns, academic stress and socioeconomic status were not considered in the questionnaire. Fourth, the effect sizes of the individual dietary exposures (e.g., sugar-sweetened beverages, milk tea, coffee) were modest, with some lower confidence intervals close to 1.0, indicating limited precision. In addition, we could not directly measure pubertal stage or hormone levels, and although age was adjusted for in the models, the large OR for age (>5) suggests that residual confounding by these unmeasured biological factors may still exist. Fifth, the reference group for the Dietary Exposure Index (DEI) combined DEI = 0 and DEI = 1 due to a small sample size. This merging may have shifted the reference upward and biased the odds ratios toward the null, especially given that the distribution of DEI scores was skewed toward higher values. Despite this bias, the association between DEI and acne remained statistically significant in this study, suggesting that the true association is likely stronger than observed.

## Conclusion

This study confirmed that the intake of sugar-sweetened beverages, milk tea, and coffee is positively associated with the odds of adolescent acne, and suggests that early puberty (ages 11–13) may be a key period of interest. Therefore, these findings provide information for future analytical studies to further investigate the underlying mechanisms and temporal relationships.

## Data Availability

The raw data supporting the conclusions of this article will be made available by the authors, without undue reservation.
