# Supplementary figures and images for: The cumulative effect of multiple high-sugar and high-fat dietary patterns on acne vulgaris in school aged children: a cross-sectional study in Shanghai
Source: Front Public Health. 2026 May 8;14:1827055. doi: 10.3389/fpubh.2026.1827055 (PMC13205665; doi:10.3389/fpubh.2026.1827055)

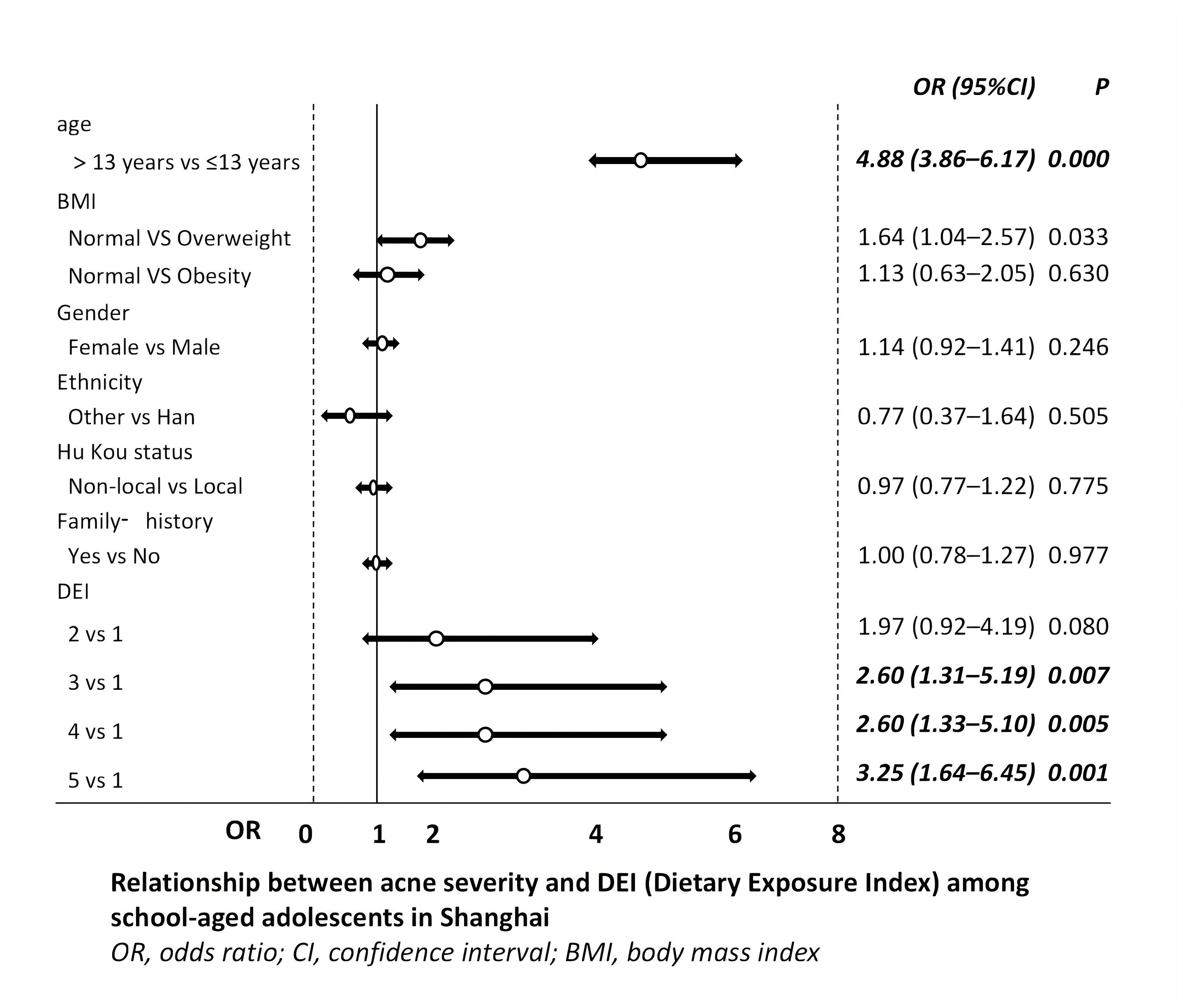

Supplement: Supplementary file 1 [file Image_1.png]
