# Supplementary material for: The cumulative effect of multiple high-sugar and high-fat dietary patterns on acne vulgaris in school aged children: a cross-sectional study in Shanghai
Source: Front Public Health. 2026 May 8;14:1827055. doi: 10.3389/fpubh.2026.1827055 (PMC13205665; doi:10.3389/fpubh.2026.1827055)
Supplement: Supplementary file 2 [file Table_1.docx]

Table 1s. Baseline characteristics of school-aged adolescents in Shanghai by acne severity.

| Characteristics | Total (n=1299) | Healthy(n=462) | Mild (n=624) | MS (n=213) | **P** |
| --- | --- | --- | --- | --- | --- |
| **Age, median (P25, P75)** | 13 (13,14) | 13 (12,13) | 14 (13,14) | 14 (13,15) | 0.000 |
| **Age group, n(%)** |  |  |  |  | 0.000 |
| ≤13 years | 715 (55.0) | 372 (52.0) | 279 (39.0) | 64 (9.0) |  |
| >13 years | 584 (45.0) | 90 (15.4) | 345 (59.1) | 149 (25.5) |  |
| Height (cm), mean (SD) | 164.0 (8.9) | 160.6 (8.7) | 165.7 (8.8) | 166.1 (7.3) | 0.000 |
| Weight (kg), mean (SD) | 53.9 (13.1) | 50.1 (12.2) | 55.9 (13.7) | 56.2 (11.4) | 0.000 |
| BMI (kg/m^2^), mean (SD)^a^ | 19.9(4.0) | 19.3(4.0) | 20.2 (4.1) | 20.3 (3.5) | 0.000 |
| **BMI group, n(%)** |  |  |  |  | 0.012 |
| N**ormal (<24.0)** | 1179 (90.8) | 436 (37.0) | 550 (46.6) | 193 (16.4) |  |
| Overweight (24.0-27.9) | 77 (5.9) | 17 (22.1) | 46 (59.7) | 14 (18.2) |  |
| Obesity (≥28.0) | 43 (3.3) | 9 (20.9) | 28 (65.1) | 6 (14.0) |  |
| **Grade, n(%)** |  |  |  |  | 0.000 |
| Six | 364 (28.0) | 223 (61.3) | 121 (33.2) | 20 (5.5) |  |
| Seven | 328 (25.3) | 154 (47.0) | 127 (38.7) | 47 (14.3) |  |
| Eight | 404 (31.1) | 69 (17.1) | 258 (63.9) | 77 (19.0) |  |
| Nine | 203 (15.6) | 16 (7.9) | 118 (58.1) | 69 (34.0) |  |
| **Gender, n(%)** |  |  |  |  | 0.446 |
| Male | 688 (53.0) | 252 (36.6) | 331 (48.1) | 105 (15.3) |  |
| Female | 611(47.0) | 210 (34.4) | 293 (47.9) | 108 (17.7) |  |
| **Ethnicity, n(%)^b^** |  |  |  |  | 0.515 |
| Han | 1272 (97.9) | 454 (35.7) | 607 (47.7) | 211 (16.6) |  |
| Other Ethnicity | 27 (2.1) | 8 (29.6) | 17 (63.0) | 2 (7.4) |  |
| **Hu Kou status, n(%)^c^** |  |  |  |  | 0.003 |
| Local | 830 (63.9) | 267 (32.2) | 418 (50.4) | 145 (17.4) |  |
| Non-local | 469 (36.1) | 195 (41.6) | 206 (43.9) | 68 (14.5) |  |
| ****Family history, n(%)**** |  |  |  |  | 0.001 |
| No | 960 (73.9) | 333 (34.7) | 488 (50.8) | 139 (14.5) |  |
| Yes | 339 (26.1) | 129 (38.1) | 136 (40.1) | 74 (21.8) |  |

MS, moderate and severe; BMI, body mass index; SD, standard deviation;

Table 2s. Dietary Exposures of School-Aged Adolescents in Shanghai by Acne Severity Groups

| Dietary | Total (n=1299) | Healthy (n=462) | Mild (n=624) | MS (n=213) | P |
| --- | --- | --- | --- | --- | --- |
| Dairy products, n (%) | |  |  |  | 0.897 |
| Yes | 1209 (93.1) | 432 (35.7) | 579 (47.9) | 198 (16.4) |  |
| No | 90 (6.9) | 30 (33.3) | 45 (50.0) | 15 (16.7) |  |
| SSB, n (%) |  |  |  |  | 0.046 |
| Yes | 1130 (87.0) | 388 (34.3) | 556 (49.2) | 186 (16.5) |  |
| No | 169 (13.0) | 74 (43.8) | 68 (40.2) | 27 (16.0) |  |
| Milk tea, n (%) | |  |  |  |  |
| Yes | 1005 (77.4) | 342 (34.0) | 484 (48.2) | 179 (17.8) | 0.015 |
| No | 294 (22.6) | 120 (40.8) | 140 (47.6) | 34 (11.6) |  |
| Coffee, n (%) |  |  |  |  | 0.000 |
| Yes | 432 (32.4) | 115 (27.3) | 222 (52.7) | 84 (20.0) |  |
| No | 878 (67.6) | 347 (39.5) | 402 (45.8) | 129 (14.7) |  |
| Bakery Products, n (%) | |  |  |  | 0.228 |
| Yes | 1133 (87.2) | 403 (35.6) | 537 (47.4) | 193 (17.0) |  |
| No | 166 (12.8) | 59 (35.5) | 87 (52.4) | 20 (12.1) |  |
| DEI, n (%)^a^ |  |  |  |  | 0.000 |
| 1 | 39 (3.0) | 20 (51.3) | 18 (46.1) | 1 (2.6) |  |
| 2 | 103 (7.9) | 44 (42.7) | 46 (44.7) | 13 (12.6) |  |
| 3 | 291 (22.4) | 101 (34.7) | 144 (49.5) | 46 (15.8) |  |
| 4 | 546 (42.0) | 214 (39.2) | 242 (44.3) | 90 (16.5) |  |
| 5 | 320 (24.6) | 83 (25.9) | 174 (54.4) | 63 (19.7) |  |

SSB, Sugar-sweetened beverages;MS,Moderate and Severe

^a^ DEI (Dietary Exposure Index): Cumulative score (0–5) for five items: dairy products, sugar-sweetened beverages, milk tea, coffee, and bakery products (1 point per item). DEI=0 (n=4) was merged into the DEI=1 group to form the low-exposure reference.


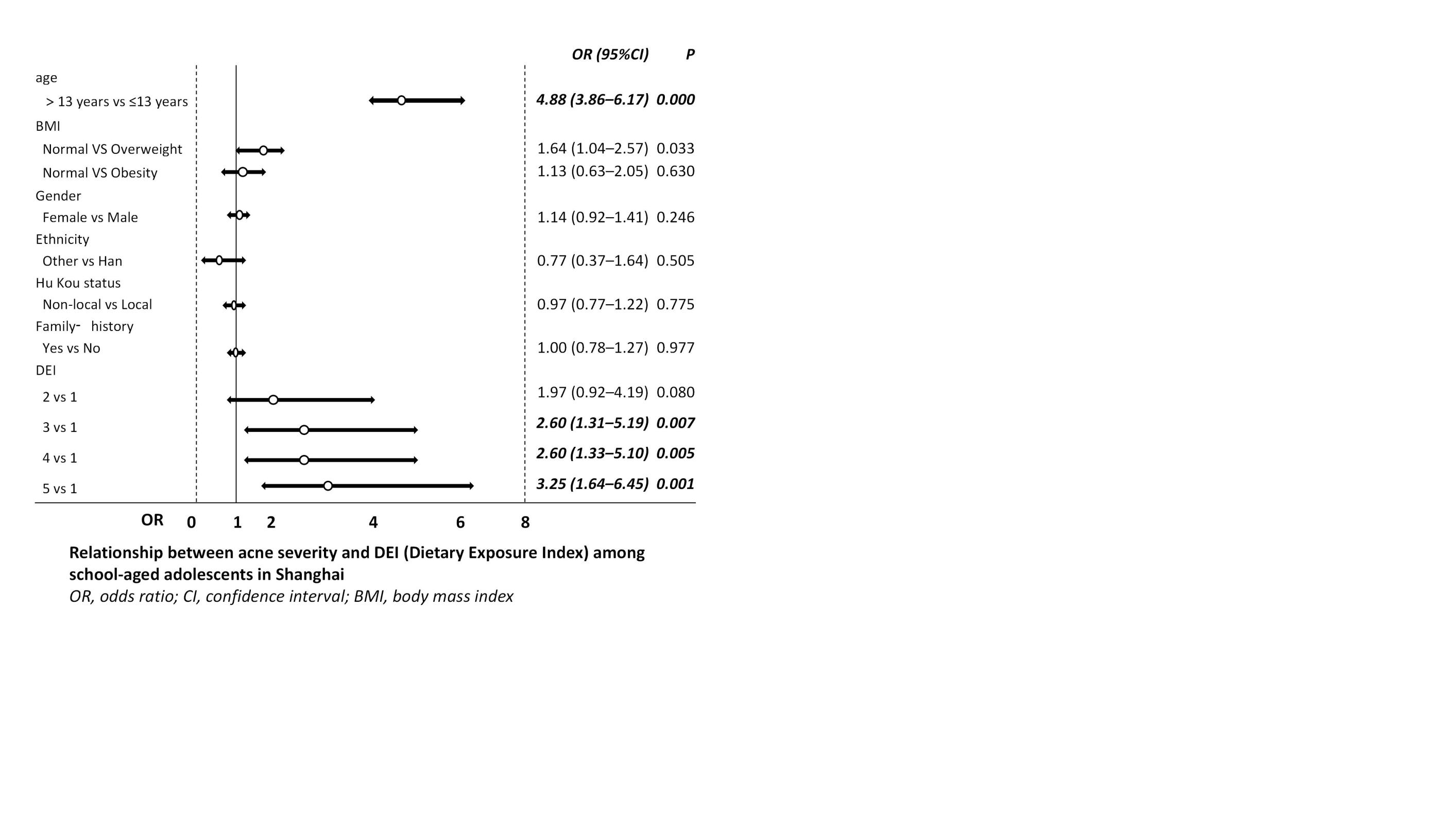


Figure 1s. Association of age, BMI, household registration status, and dietary exposure index (DEI) with acne severity (moderate and severe, mild vs healthy) among school-aged adolescents in Shanghai: results from logistic regression analysis.
